# Nervous System Development and Neuropeptides Characterization in Embryo and Larva: Insights from a Non-Chordate Deuterostome, the Sea Cucumber *Apostichopus japonicus*

**DOI:** 10.3390/biology11101538

**Published:** 2022-10-20

**Authors:** Yingqiu Zheng, Xiao Cong, Huachen Liu, Yixin Wang, Kenneth B. Storey, Muyan Chen

**Affiliations:** 1 The Key Laboratory of Mariculture, Ministry of Education, Ocean University of China, Qingdao 266003, China; 2 Institute of Biochemistry, Carleton University, 1125 Colonel By Drive, Ottawa, ON K1S 5B6, Canada

**Keywords:** nervous system, neuropeptides, sea cucumber, embryo, larva

## Abstract

**Simple Summary:**

Pentamerous radial symmetrical echinoderm adults develop from bilaterally symmetrical larvae and are a great model for understanding the origin and evolution of deuterostome nervous systems. Neuropeptides are important neuronal signaling molecules that regulate diverse behavioral and physiological processes in animals including echinoderms. In this study, we revealed the remarkable complexity of embryonic and larval nervous systems, identified the neuropeptide profile, and quantified the expressions of specific neuropeptide precursor genes in *Apostichopus japonicus* embryo and larvae. Collectively, this research will enable us to have a more comprehensive understanding of the holothuroid embryonic and larval nervous system and gain insights into the potential functions of neuropeptidergic systems in holothuroid larvae.

**Abstract:**

Here, we described the complex nervous system at five early developmental stages (blastula, gastrula, auricularia, doliolaria and pentactula) of a holothurian species with highly economic value, *Apostichopus japonicus*. The results revealed that the nervous system of embryos and larvae is mainly distributed in the anterior apical region, ciliary bands or rings, and the feeding and attachment organs, and that serotonergic immunoreactivity was not observed until the embryo developed into the late gastrula; these are evolutionarily conserved features of echinoderm, hemichordate and protostome larvae. Furthermore, based on available transcriptome data, we reported the neuropeptide precursors profile at different embryonic and larval developmental stages. This analysis showed that 40 neuropeptide precursors present in adult sea cucumbers were also identified at different developmental stages of embryos and larvae, and only four neuropeptide precursors (*SWYG precursor 2, GYWKDLDNYVKAHKT precursor, Neuropeptide precursor 14-like precursor, GLRFAmprecursor-like precursor*) predicted in adults were absent in embryos and larvae. Combining the quantitative expression of ten specific neuropeptide precursor genes (NPs) by qRT-PCR, we revealed the potential important roles of neuropeptides in embryo development, feeding and attachment in *A. japonicus* larvae. In conclusion, this work provides novel perspectives on the diverse physiological functions of neuropeptides and contributes to understanding the evolution of neuropeptidergic systems in echinoderm embryos and larvae.

## 1. Introduction

Echinoderms have attracted attention from many scholars because of their key evolutionary status, strong regenerative ability, and morphological diversity [1]. Adult echinoderms, which display a radially symmetric body and radial nervous systems, have often been viewed as atavistic. The development of their body plan is closely associated with the development of the nervous system [2]. The central nervous system of adult echinoderms includes radial nerve cords (RNCs) and a circumoral nerve ring (CNR) in the oral (mouth) region [3,4]. In most echinoderms, RNCs can be further subdivided into ectoneural and hyponeural systems [3,5]. The peripheral nervous system (enteric nervous system, connective tissue plexus, and the neural circuitry of the podia or arm) that connects the RNC with other organs, is considered to be the inner entoneural system [3,6,7,8]. In echinoids, holothuroids, asteroids, and ophiuroids, the ecto and hyponeural components are dominant whereas in crinoids the inner entoneural system is the main part of the adult nervous system [8,9].

Unlike their pentaradial adults, the echinoderm larval nervous system usually shows bilateral serotonergic neurons and nerve tracts along the ciliary bands, serotonin-positive cells first appear at the gastrula stage in holothuroids, echinoids, asteroids and ophuiroids, playing a role in embryogenesis and swimming [2,10,11,12,13,14]. Nerve structures, such as neurons located in the apical organ and tracts of axons associated with the ciliary bands were identified during the larval development stage in echinoderms, which matches reports for hemichordate larvae [12,15,16]. In recent years, the development of genomic resources and molecular methods have allowed great progress for understanding the mechanisms of neurogenesis in the larvae of many echinoderm species, including the echinoids *Strongylocentrotus purpuratus*, *Paracentrotus lividus*, the asteroids *Patiria miniata*, *Asterias rubens*, the ophiuroids *Amphipholis kochii*, the crinoids *Antedon mediterranea*, *Metacrinus rotundus*, *Anneissia japonica*, and also the holothuroids *Apostichopus californicus*, *Apostichopus parvimensis* and *Apostichopus japonicus* [2,13,17,18,19,20,21,22]. These studies all indicated that the larval nervous system shows unexpected diversity in cell and fiber types and their distribution in both central and peripheral nervous components [2,13,17,18,19,20,21,22]. Among echinoderms, the planktotrophic holothuroid sea cucumbers retain both the ancestral body plan and the ancestral nervous system developmental pattern of echinoderms: a lack of neural precursor migration in the embryo and a feeding initiation stage-auricularia followed by a doliolaria stage [11,23]. *Apostichopus japonicus*, a classical planktotrophic sea cucumber, has multiple larval strategies and its life cycle can be divided into eight major stages: fertilization (0 hpf (hours post fertilization)), blastula (14 hpf), gastrula (24 hpf), auricularia (48 hpf), doliolaria (11 dpf (days post fertilization)), pentactula (12 dpf), juvenile (16 dpf), and adult [24]. Although some advances have been made to elucidate certain developmental stages of the holothuroids’ nervous system [2,21,25], a comprehensive study of the nervous system from the blastula to pentactula stages has still not been conducted.

Neuropeptides are considered to be the oldest neuronal signaling molecules in metazoans, which makes them optimal tracers for neuroendocrine activity [26,27,28]. Studies of marine invertebrate neuropeptide systems have been revealed in Mollusca, annelids, marine arthropods (crustaceans) and echinoderms [29,30,31,32,33,34,35,36,37,38,39]. In echinoderms, the recent development of RNA high-throughout sequencing technology has allowed strong advances in the identification and characterization of neuropeptides in adults [40,41,42,43,44,45,46,47,48,49]. Neuropeptides in adult echinoderms have been proven to play important roles in muscle contractility, feeding and reproduction [50,51,52,53,54,55,56,57,58,59,60,61,62]. In comparison with adults, little is known about neuropeptide localization and function during the larval stages of most echinoderms, especially in holothuroids. Limited studies of echinoids and asteroids indicated the potential function of neuropeptides in larval locomotion, feeding, digestive system, attachment, and metamorphosis [19,52,63,64].

The foundation for the present study was our recent identification of 44 neuropeptide precursor transcripts in the CNR of adult *A. japonicus*, which represents the most comprehensive resource to date for sea cucumber neuropeptide research [48]. However, the anatomy and nervous system of larvae are completely different from adult animals. Hence, we aimed first to investigate the comprehensive landscape of the nervous system from blastula to pentactula by immunofluorescence (IF). Secondly, we aimed to describe whether neuropeptide precursors predicted in the adult nervous system were also expressed in sea cucumber embryos and larvae by analyzing the published transcriptome database at different developmental stages, and further explore the expression of ten specific neuropeptides precursor genes (NPs): *A. japonicus Kisspeptin-type precursor* (*AjKPP*), *A. japonicus Gonadotropin-releasing hormone*-*type precursor* (*AjGnRHP*), *A. japonicus Calcitonin-type precursors* (*AjCTP1*/*2*: *AjCTP1* and *AjCTP2*), *A. japonicus MPMNPADYFSRGTVYIPTRDS precursor* (*AjMS21P*), *A. japonicus Pedal peptide-type precursor 2* (*AjPPLNP2*), *A. japonicus Vasopressin/oxytocin-type precursor* (*AjholotocinP*), *A. japonicus Thyrotropin-releasing hormone* (*TRH*)-*type precursor* (*AjTRHP*), *A. japonicus Bursicon alpha-type precursors* (*AjBAP*), and *A. japonicus Orexin-type precursors* (*AjOXP1* and *AjOXP2*) by quantitative real-time PCR (qRT-PCR). Nine of the NPs that we selected, except *AjMS21P*, were chosen because they have been reported in other echinoderms previously and our group has performed functional research on these and proven their importance in biological processes in adult sea cucumbers (unpublished data). The remaining NP (*AjMS21P*) has only been identified in *A. japonicus* to date and possibly plays important roles in adult sea cucumbers [48]. The present research will enable us to have a more comprehensive understanding of the embryonic and larval holothuroid nervous system and gain insights into the potential functions of neuropeptide systems in sea cucumber embryos and larvae.

## 2. Materials and Methods

### 2.1. Embryo and Larval Culture

Typical early period embryos and larvae of *A. japonicus* (blastula (14 hpf), gastrula (24 hpf), late-gastrula (34 hpf), auricularia (48 hpf), doliolaria (11 dpf), and pentactula (12 dpf)) specimens were collected from Shandong oriental ocean sea cucumber breeding farm (Weihai, China) in May 2020. The embryos and larvae were cultured in filtered sea water (temperature: 20–21 °C, salinity: 32 ppt, dissolved oxygen level: 8 mg/L) under the density of 0.5~0.8 individual/ml (ind/mL) and all specimens were collected using a 60 μm filter. For immunostaining, the specimens were fixed in 4% paraformaldehyde (PFA) in 0.1 M phosphate-buffered saline (PBS) for 15 min at room temperature (RT) and were washed four times in PBS. The animals were dehydrated through a graded methanol series and then transferred into ice-cold methanol and stored at −20 °C until use [2,65]. For qRT-PCR analysis, the filtered samples (six biological replicates) were frozen in liquid nitrogen and stored at −80 °C for use. All animal care and use procedures were approved by the Institutional Animal Care and Use Committee of Ocean University of China (Permit Number: 20141201) and performed according to the Chinese Guidelines for the Care and Use of Laboratory Animals (GB/T 35892-2018).

### 2.2. Immunostaining

For nervous system immunostaining of echinoderms, acetylated α-tubulin present in axons and dendrites and neurotransmitter serotonin were used as primary antibodies in the present study [66,67,68,69,70]. Fixed *A. japonicus* larvae were transferred onto adhesion microscope slides (Citotest) and dried at 25 °C for 10 mins. Specimens were then circled with a Liquid Blocker pen and incubated with 3% H_2_O_2_ resolutions at room temperature (RT) for 25 min to pre-block. Blocking was carried out using 5% goat serum (Solarbio, Cat# SL038) for 30 min at RT followed by subsequent incubation with primary antibodies in PBST overnight at 4 °C. The primary antibodies were: mouse anti-acetylated α-tubulin (Sigma-Aldrich, Cat# T6793) used at 1:200 and rabbit anti-serotonin (ImmunoStar, Cat# 20080) used at 1:200. After incubation, primary antibodies were removed by three washes in PBST for 10 mins each at RT. Specimens were then incubated for 1 hour at RT with one of two secondary antibodies diluted 1:600 in PBS: Alexa Fluor 488-AffiniPure Goat Anti-Rabbit IgG (H+L) (Jackson, Cat# 111-545-003) or Rhodamine (TRITC)–conjugated Goat Anti-Mouse IgG(H+L) (Proteintech, Cat# SA00007-1). Nuclear staining was performed for 10 mins at RT using DAPI (Solarbio, Cat# C0065). To test the specificity of the antibodies, negative control treatments were carried out by omission of the primary antibody (Supplementary Figure S1). All immunostaining was imaged using a Fluorescence microscope system (Olympus BX53F).

### 2.3. Identification of Neuropeptide Precursors and Putative Neuropeptides in A. japonicus Embryonic and Larval Stages

The transcriptomes of *A. japonicus* at different developmental stages (blastula, gastrula, auricularia, pentactula) were downloaded from the NCBI database (accession numbers: SRR6075435-SRR6075438). As the original source reported, one replicate per stage (~100 embryos/larvae) was used for transcriptome sequencing, resulting in 230.8 million raw paired-end reads [71]. Low-quality reads were filtered using Trimmomatic v0.39 with the following parameters: “LEADING:30 TRAILING:30 SLIDINGWINDOW:5:30 AVGQUAL:34 MINLEN:21” [72]. The clean reads generated were applied for constructing de novo assemblies using Trinity v2.12.0 [73,74]. Finally, we obtained a total of 133,040, 172,308, 158,993, 134,712 transcripts in blastula, gastrula, auricularia and pentactula respectively and these four larval transcriptome libraries were used for local BLAST with Protein Query-Translated Subject BLAST (Version 2.12.0+). The data that support the findings of this study have been deposited into the CNGB Sequence Archive (CNSA) of the China National GeneBank DataBase (CNGBdb) [75,76] with accession number CNP0002851. To search for transcripts encoding putative neuropeptide or peptide hormone precursor proteins at different developmental stages, the sequences of neuropeptides or peptide hormone precursors previously identified in *A. japonicus* [48] were submitted individually as queries in a local blast search of the four transcriptome databases using Protein Query Translated Subject BLAST (Version 2.12.0+) with the e-value setting set to 0.01.

### 2.4. RNA Isolation, cDNA Synthesis and Full-Length Cloning of Putative Neuropeptide Precursor Genes (NPs)

Total RNA was isolated from *A. japonicus* embryos and larvae using Trizol (Takara, Japan, Code # 9109) according to the manufacturer’s instructions, and the RNA quality was determined via spectrophotometry using a NanoDrop 2000 (Thermo, Waltham, MA, USA) and 1% agarose gel electrophoresis. The full-length cDNA sequences encoding *AjTRHP*, *AjPPLNP2*, *AjMS21P*, *AjCTP1*, *AjCTP2* and *AjHolotocinP* were amplified using a SMARTer^®^ RACE 50/30 Kit (Clontech, Mountain View, CA, USA, Cat # 634858) as described in Wang et al. (2019) [77] and sequenced by BGI TECH SOLUTIONS (BEIJING LIUHE) CO., LIMITED Qingdao, China. Primer information is listed in Supplementary Table S1.

### 2.5. Quantitative Real-Time PCR (qRT-PCR) in Early Developmental Stages of A. japonicus

The samples at five stages were collected and RNA from six biological replicates (6 × 10^3^–7 × 10^3^ individuals/replicate per stage) was isolated. Relative transcript levels were determined using a TB Green^®^ Premix Ex Taq™ (Tli RNaseH Plus) (Takara, Cat# RR420A) with a StepOnePlus (ABI Inc., Foster City, CA, USA). Each sample was run in triplicate. The specific primers for NPs were designed using Primer 6 software (Version 5.0) and are listed in the Supplementary Table S1. A special case is that of *AjCTP1*, which has only one more short-fragment exon as compared with *AjCTP2*, making it difficult to design specific primers to distinguish them. Therefore, the common part of the two sequences was applied to design a specific primer for detecting the total relative transcript levels of *AjCTP1/2* (*AjCTP1* + *AjCTP2*). *β*-actin (ACTB, PIK61412.1) and *β*-Tubulin (TUBB, PIK51093) were used as housekeeping genes for standardization, as previously validated [78]. The 2^−ΔΔCT^ method was applied to analyze the comparative expression levels. All data are given as the mean ± S.E. (*n* = 6) and were analyzed using a one-way analysis of variance (ANOVA) followed by a Tukey post hoc test (SPSS 17.0, Inc., Chicago, IL, USA). The level of statistical significance was set at *p* < 0.05.

## 3. Results

### 3.1. Nervous System Profile of A. japonicus at Different Developmental Stages

**Figure 1:**
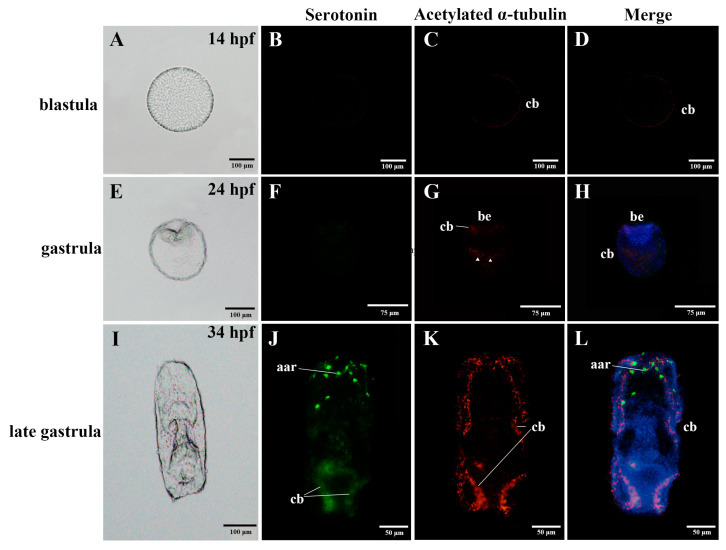
Anatomy diagrams and localization of the nervous system in the embryos of the sea cucumber *A. japonicus*, using immunofluorescence. (**A**–**D**) blastula; (**E**–**H**) gastrula; (**I**–**L**) late gastrula. Labels are: aar, anterior apical region; be, blastopore; cb, ciliary band. Triangles on (**G**) label the positive immunoreactions in the bottom half of the gastrula. Green, serotonin; Red, acetylated α-tubulin. Information of hpf (hours post fertilization) was labelled on the upper right of the anatomy diagrams.

**Figure 2:**
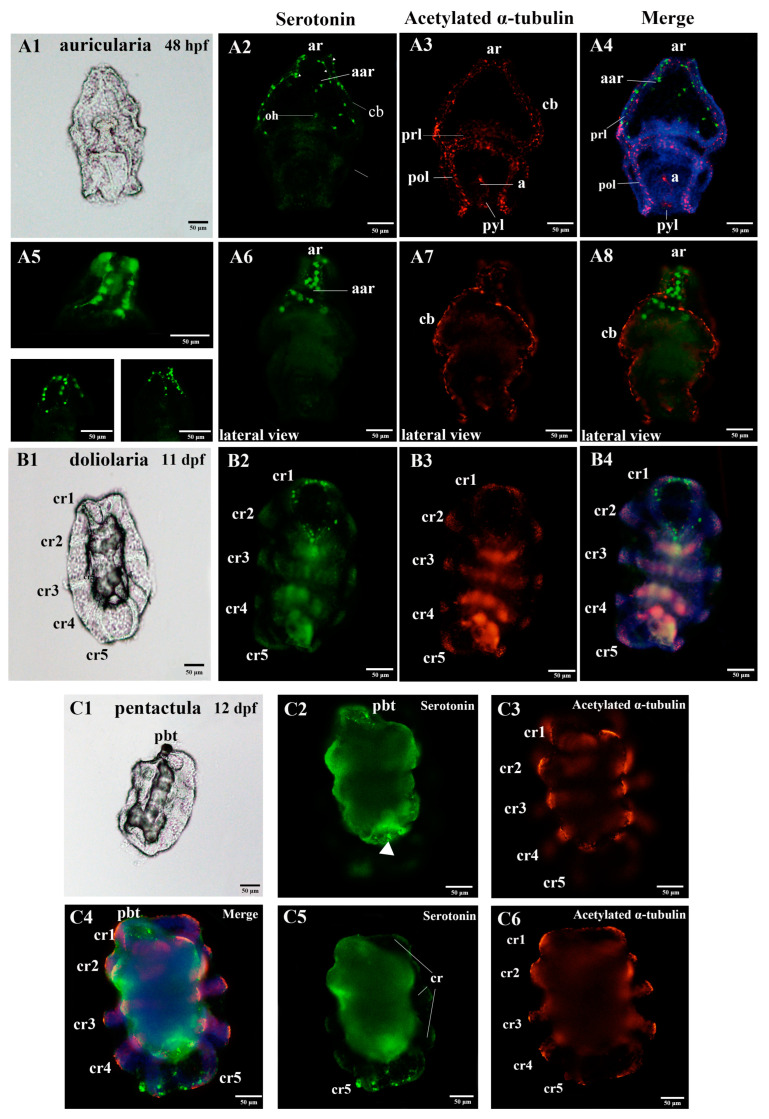
Anatomy diagrams and localization of the nervous system in the larvae of the sea cucumber *A. japonicus*, using immunofluorescence. (**A1**–**A8**) auricularia; (**B1**–**B4**) doliolaria; (**C1**–**C6**) pentactula. (**A5**) is a high resolution picture of the anterior region of auricularia stained for serotonin (green). (**A6**–**A8**) are lateral views of the serotonin and acetylated α-tubulin staining. (**C2**,**C3**,**C5**,**C6**) are pictured by focusing on the different layers, respectively. The labels are: a, anus; aar, anterior apical region; ar, apical ridge; cb, ciliary band; cr, ciliary ring; oh, oral hood; pbt, primary buccal tentacle; pol, posterior loop; prl, preoral loop; pyl, pylorus. Green, serotonin; Red, acetylated α-tubulin. Information of hpf (hours post fertilization) and dpf (days post fertilization) is labelled at the upper right of the anatomy diagrams.

**Figure 3:**
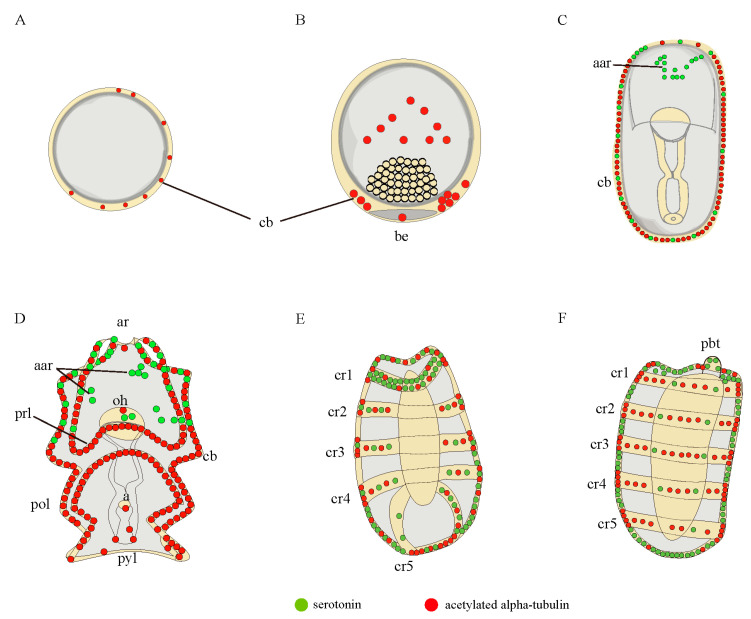
A diagrammatic representation of the nervous system of *A. japonicus* embryos and larvae stained by serotonin (green) and acetylated α-tubulin (red). (**A**) blastula; (**B**) gastrula; (**C**) late gastrula; (**D**) auricularia; (**E**) doliolaria; (**F**) pentactula. The labels are: a, anus; aar, anterior apical region; ar, apical ridge; be, blastopore; cb, ciliary band; cr, ciliary ring; oh, oral hood; pbt, primary buccal tentacle; pol, posterior loop; prl, preoral loop; pyl, pylorus.

To acquire a comprehensive characterization of the nervous system of *A. japonicus* over the developmental stages of embryos and larvae, we applied two regularly used metazoan nervous system markers, acetylated α-tubulin and serotonin, to profile the nervous system of *A. japonicus* by IF (Figure 1, Figure 2 and Figure 3) [13,21]. The embryo and larvae stages were divided according to Qiu et al. (2015) [24]. Names of the five early developmental stages (blastula, gastrula, auricularia, doliolaria, pentactula) body parts are as described in Figure 1, Figure 2 and Figure 3, and negative controls were performed and are shown in Supplementary Figure S1.

At the embryonic stage, serotonin-immunoreactions were not observed until the embryos developed into late-gastrula, where they were located in the anterior apical region, and along the ciliary bands (Figure 1B,D,F,H,J,L and Figure 3A–C). Expressions of acetylated α-tubulin were detected in the ciliary band at the blastula and late-gastrula stages, and were also identified in the ciliary band, blastopore and in the bottom half of the gastrula (Figure 1C,D,G,H,K,L and Figure 3A–C). Serotonin and acetylated α-tubulin were co-expressed in the ciliary band (Figure 1L and Figure 3C).

At the larvae stages, the auricularia larvae possessed two clusters of bilaterally serotonergic staining along the ciliary bands in the apical region and projected a fine axon-like structure in the front and lateral view (Figure 2A2,A5,A6 and Figure 3D). We also observed positive serotonergic staining around the oral hood (Figure 2A2 and Figure 3D). After the transition from auricularia to doliolaria, the ciliary band nerve tracts gradually moved with the rearranged ciliary bands and remained immunoreactive (Figure 2B2,B4 and Figure 3E). Positive serotonergic nerve immunoreactions were observed in five ciliary rings and formed a circle-like structure in the first ciliary ring (Figure 2B2,B4 and Figure 3E). When the doliolaria larvae developed into an early pentactula stage, the serotonergic immunoreactivity was detected in the ciliary rings, especially in the fifth ciliary ring and primary buccal tentacle (Figure 2C2,C4,C5 and Figure 3F).

In addition, acetylated α-tubulin staining was observed mainly over the ciliary band, pre-oral loop, and post-oral loop in auricularia. Positive immunoreactions were also identified in the apical ridge and around the anus and pylorus (Figure 2A3,A4,A7,A8 and Figure 3D). With the transformation in morphology, acetylated α-tubulin protein was clearly identified in the ciliary rings in the doliolaria and early pentactula stages (Figure 2B3,B4,C3,C4,C6 and Figure 3E,F). Colocalization of serotonin and acetylated α-tubulin were observed in the ciliary band and anterior apical region in auricularia, and ciliary rings in doliolaria and pentactula (Figure 2A4,A8,B4,C4 and Figure 3E,F).

### 3.2. Identification of Neuropeptide Precursor Transcripts in A. japonicus Embryo and Larvae

**Figure 4:**
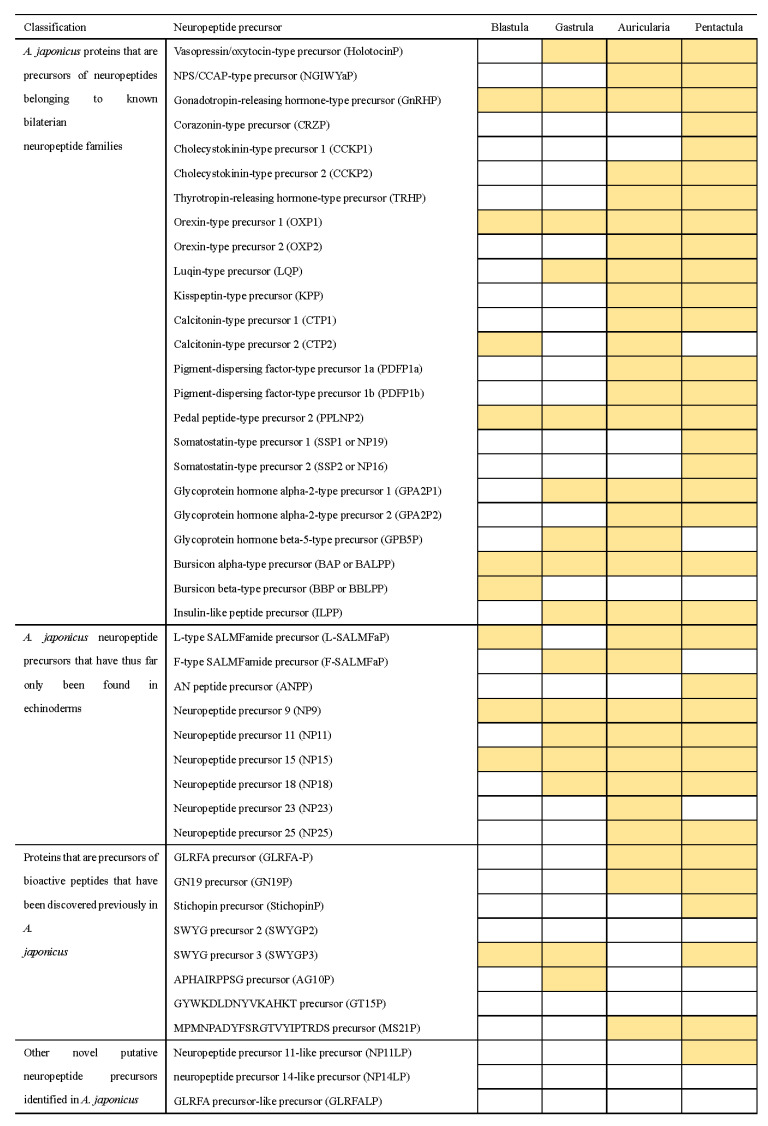
Blast-based identification of neuropeptide precursors in *A. japonicus* embryos and larvae. The shading indicates that the transcripts of NP were identified.

A total of 44 neuropeptide precursor transcripts predicted in adult *A. japonicus* [48] were submitted as queries for in silico tBLASTn analysis based on the transcriptomes of *A. japonicus* larvae from four developmental stages with the following accession numbers: SRR6075437 (blastula), SRR6075438 (gastrula), SRR6075435 (auricularia), and SRR6075436 (pentactula) with the e-value setting set to 0.01 [71]. The results showed that the transcriptional expression pattern of neuropeptide precursors is specific in different developmental stages (Figure 4), and the identity percentage of the hits for the results of tBLASTn-based analysis is shown in the Supplementary Data 1. For NPs belonging to known bilaterian neuropeptide families, only four neuropeptides were expressed in all four early developmental stages, whereas the *Cholecystokinin-type precursor1* (*CCKP1*), *Somatostatin-type precursors* (*SSP1* and *SSP2*) and *Corazonin-type precursor* (*CRZP*) were only present at the pentactula stage. One third of the NPs were not transcribed until the larvae developed into the auricularia stage, and *Bursicon beta-type precursors* (*BBP*) were only found in blastula. *NP9* and *NP15* of *NPs* that have, thus far, been expressed as specific to echinoderms appeared at all four stages. *L-type SALMFamide precursor* (*L-SALMFaP*) was absent only in gastrula larvae; whereas *F-type SALMFamide precursor* (*F-SALMFaP*) was absent at the blastula and pentactula stages. Some NPs, such as *AN peptide precursor* (*ANPP*) and *NP23*, were present only at the pentactula or auricularia stages. *NP25* were not transcribed at the first two stages (blastula and gastrula). For *NPs* that were previously discovered in *A. japonicus*, none of the NPs were present at all four stages and the *GLRFA precursor* (*GLRFA-P*), *GN19 precursor* (*GN19P*) and *MS21P* were found at the late developmental stages (auricularia and pentactula stages). *StichopinP* was only expressed in the pentactula stage. Other novel putative neuropeptide precursors were found only when *neuropeptide precursor 11-like precursor (NP11LP)* were expressed in pentactula. Interestingly, four neuropeptide precursors (*SWYGP2*, *GT15P*, *NP14LP*, *GLRFALP*) predicted in adults were absent in embryos and larvae.

### 3.3. Quantitative Analysis of Specific Neuropeptide Precursors in A. japonicus Embryos and Larvae

**Figure 5:**
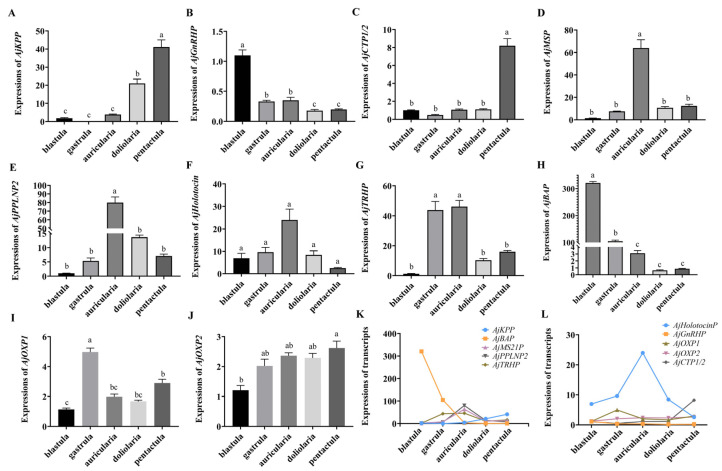
Relative transcriptional level of NP genes in the embryos and larvae of the sea cucumber *A. japonicus* revealed by quantitative real-time PCR (qRT-PCR). (**A**) *AjKPP*; (**B**) *AjGnRHP*; (**C**) *AjCTP1/2*; (**D**) *AjMS21P*; (**E**) *AjPPLNP2*; (**F**) *AjHolotocinP*; (**G**) *AjTRHP*; (**H**) *AjBAP*; (**I**) *AjOXP1*; (**J**) *AjOXP2;* (**K**) Comprehensive analysis of five NP genes (*AjKPP, AjBAP, AjMS21P, AjPPLNP2, AjTRHP*) expression; (**L**) Comprehensive analysis of five NP genes (*AjHolotocinP, AjGnRHP, AjOXP1*, *AjOXP2, AjCTP1/2*) expression. Different lowercase letters indicate significant differences between the different stages (*p* < 0.05).

The expression levels of NP genes in the embryonic (blastula, gastrula) and larvae (auricularia, doliolaria, and pentactula) stages (six biological replicates) were detected using quantitative real-time PCR (qRT-PCR) (Figure 5). The ten NP genes were present with different expression patterns, and are basically consistent with those in the transcriptome. Few expressions of *AjKPP* were detected in the first three stages, which was significantly lower than that in the doliolaria and pentactula (*p* < 0.05) (Figure 5A), and *AjGnRHP* was significantly highly expressed in the blastula stage (*p* < 0.05) (Figure 5B). *AjCTP1/2* was barely expressed until the larvae developed into thee pentactula stage (Figure 5C). *AjMS21P* (Figure 5D) and *AjPPLNP2* (Figure 5E) had a dominant expression in auricularia larvae, which was obviously higher than that at the gastrula, doliolaria and pentactula stages (*p* < 0.05). Similar with *AjPPLNP2* and *AjMS21P*, *AjHolotocinP* had a peak expression at the auricularia stage, but there was no difference among the five stages (*p* > 0.05) (Figure 5F). For *AjTRHP*, the expression levels at the gastrula and auricularia stages were significantly higher than that at the blastula, doliolaria and pentactula stages *(p* < 0.05) (Figure 5G). *AjBAP* was significantly highly expressed in embryos (blastula and gastrula) than in larvae, especially in the blastula (Figure 5H). *AjOXP1* and *AjOXP2* showed different expression pattern. *AjOXP1* had the highest expression at the gastrula stage and the lowest expression at the blastula stage (Figure 5I), whereas expression of *AjOXP2* at the pentactula stage was significantly higher than those at the blastula stage (Figure 5J). Overall, ten NP genes showed a complex transcriptional pattern (Figure 5K,L).

## 4. Discussion

### 4.1. Nervous System Complexity at Early Developmental Stages 

Serotonergic neurons are the first neurons to differentiate in most echinoderm and hemichordate species and are thought to be primary sensory neurons with short apical dendritic poles and basal axonal projections [12,16]. In the late gastrula stage, we observed serotonin-positive immunoreactions in the anterior apical region, that matches the discovery in echinoderms, hemichordates, and protostomes with swimming larvae [2,12,13,18,79,80,81]. Interestingly, serotonergic immunoreactions were also identified in the ciliary band of *A. japonicus* embryos and larvae, supporting the potential role of serotonin in the modulation of ciliary beating and metamorphosis. Similarly, the nervous system of echinoderm larvae, like those of the starfish *Asterina pectinifera* and the sea urchin *S. purpuratus*, appears to be centered on the ciliary band, whose function may be to sense environmental cues [15,82,83].

Unlike asteroids [13,16], early serotonergic cells do not migrate in the auricularia as the ciliary band forms and a serotonergic nerve tract connects the left and right ciliary band tracts at the apical ridge of the auricularia in holothurian species [2,25] including in our present study. Ciliary bands change into ciliary rings during the transformation from auricularia to doliolaria and retain immunostaining in *A. japonicus*. Consistent with the previous study in *A. japonicus* [2], the rearrangement of ciliary bands followed the rearrangement of the larval nervous system. It was clearer in the larvae stained by an acetylated α-tubulin antibody, which is widely used as a pan-neuronal marker labeling neurites and cilia [69,70,84]. Acetylated α-tubulin was found in the ciliary band or ring at all developmental stages in *A. japonicus* and regularly spaced within the ciliary rings in doliolaria and early pentactula larvae, which is consistent with the dipleurula-type larvae of sea urchin, starfish, brittle star and feather star echinoderms, and evolutionarily closely related cephalochordates and hemichordates [13,18,21,69,70,85]. Our observation supports the possible principal role of the ciliary band in locomotion and feeding, which is possibly controlled by the nervous system in echinoderm larvae [16,86,87]. Nerve components were also observed in several organs, such as the oral hood, anus, and pylorus. Generally, positive staining along with the ciliary bands and around the organs, such as the oral hood, anus, and pylorus, make up the peripheral nervous system in larvae, which has also been reported in other echinoderms [12,19]. 

At the initial stage of the adult nervous system, the early pentactula stage in *A. japonicus*, we found intense serotonergic immunoreactions in the anterior-most region, which is consistent with the observations in metamorphosing larvae of the asteroid, *A. kochii*, and the pentacrinoid larvae of the crinoid *A. mediterranea* [18,85]. In the present study, we found that serotonin-immunoreactions mainly gathered at the anterior region in all investigated larvae after the late gastrula stage and were obvious in the first ciliary ring where nerve rings formed in doliolaria and the early pentactula larvae of *A. japonicus* [88,89]. This supports the hypothesis of anterior neurons as a subset of apical organ neurons that are considered to be the central nervous system of unattachment larvae [12]. Therefore, the common ancestor of echinoderms may have possessed a central integrated nervous organ during the larvae stage to regulate biological processes including development, feeding, swimming and attachments [8,13,18]. However, the detailed regulatory pattern and pathway of the larval nervous system remain to be further studied.

### 4.2. Neuropeptides at Early Developmental Stages

Neuropeptides are present across the bilaterians, suggesting that these ancient molecules play a vital role in the function and evolution of nervous systems [28,90]. Recent studies have revealed multiple neuropeptides at the early developmental stages in echinoid and asteroid species [19,64]; however, little work has been carried out on holothurians. Here, the available transcriptome data gives us a chance to report the first identification of 40 NPs at different developmental stages of the embryos and larvae of *A. japonicus*, and note that four neuropeptide precursors (*SWYGP2*, *GT15P*, *NP14LP*, *GLRFALP*) predicted in adults were absent in embryo and larvae [48]. The differences in neuropeptide variety at different developmental stages results, perhaps, from two reasons: (1) the quality of the genome and transcriptome is not good enough to make the correct assemble of transcripts; (2) the neuropeptides really do not express at this developmental stage, so it is impossible for them to play a role at this stage.

Among the NPs identified in embryos and larvae, a few of them (10/40) were expressed in the blastula, indicating that neuropeptidergic systems appear from the early stage of embryonic development. Thirteen of them were not identified until the auricularia stage of *A. japonicus*, including *NPS*/*CCAP-type precursor* (*NGIWYamide precursor, NGIWYaP*), *Cholecystokinin-type precursor 2* (*CCKP2*), *TRHP, Orexin-type precursor 2* (*OXP2*), *Kisspeptin-type precursor* (*KPP*), *Calcitonin-type precursor 1* (*CTP1*), *Pigment-dispersing factor-type precursors* (*PDFP1a* and *PDFP1b*), *Glycoprotein hormone alpha-2-type precursor 2* (*GPA2P2*), *NP25, GLRFA-P, GN19P*, *MS21P*. Our qRT-PCR analysis also revealed peak expressions of *AjTRHP*, *AjPPLNP2*, *AjHolotocinP* and *AjMS21P* at the auricularia larvae stage. (Figure 5). Auricularia is a key stage at which the larvae begin to have feeding and digestive tissues and organs [24]. Therefore, we speculated that these NPs may play an important role in the feeding process. In addition, previous studies have also reported that the expression of TRH was involved in regulating feeding behavior in sea urchin larvae [19]. Holotocin, as a member of the VP/OT family, plays ancient roles in regulating feeding and has been experimentally proven to be involved in feeding in *A. rubens* [91]. Interestingly, AjMS21 was identified as a myoactive peptide in adults [92,93,94], suggesting that it may be involved in the regulation of feeding behavior by controlling the relaxation and contraction of feeding organs throughout the sea cucumber lifecycle. All of the above further support the putative roles of these neuropeptides in regulating larval feeding.

Attachment and metamorphosis are one of the most important early life strategies in marine invertebrates including echinoderms, which are regulated by neurotransmitters and neuropeptides [8,64,95,96,97,98]. In our present study, *CRZP, Cholecystokinin-type precursor 1* (*CCKP1*), *SSP1, SSP2, ANPP, StichopinP* and *NP11LP* were found only in the pentactula stage, which indicates a potential for these NPs in the attachment of larvae. We also observed the expression of *AjCTP* and *AjKPP* in the early pentactula stage to be significantly higher than in other stages (*p* < 0.05) (Figure 5A,C). Previous studies also revealed the presence of *CTP* in the adhesive disk, which may participate in the permanent or temporary attachment of starfish *A. rubens* [64]. Therefore, the expressions of these NPs in the *A. japonicus* pentactula stage presumably reflect the potential physiological roles of these neuropeptides in mediating the process of attachment.

*AjGnRHP* and *AjBAP* showed statistically significant differences in transcript levels at the embryo stage, suggesting their potential roles in embryo development. Previous studies also indicated that GnRH improves blastula formation and the quality of embryos, further supporting our suggestion that GnRH is involved in embryo development [99,100]. It is also noteworthy that different subtypes belonging to the same NP family were mostly (7/9) identified at different larval stages, whereas *SSP1*/*2* and *PDFP1a*/*1b* were identified at the same developmental stages, which suggests the potential complex regulatory patterns of these neuropeptide families.

## 5. Conclusions

In conclusion, this study reveals the remarkable complexity of the embryonic and larval nervous system of *A. japonicus*, reported the NPs at early stages, and detected the quantitative expression of ten specific NPs in echinoderm embryos and larvae. By describing the complex nervous system in *A. japonicus* larvae, we provide new insights into the neurophysiology of echinoderm embryos and larvae. Our present study also revealed the potential roles of neuropeptides in regulating physiological activities including embryo development, feeding and attachment. Future classification of nerve cells and functional studies of neuropeptides will continue to be carried out in larvae and adult deuterostome including our echinoderm species *A. japonicus*, which will broaden our view about the diverse physiological functions of neuropeptides in these animals and contribute to our understanding of the evolution of neuropeptidergic systems.

## Data Availability

The datasets presented in this study can be found in the online repositories of the CNGB Sequence Archive (CNSA) of the China National GeneBank DataBase (CNGBdb) (CNP0002851) at https://db.cngb.org/search/project/CNP0002851/, accessed on 30 March 2022.

## Supplementary Materials

The following supporting information can be downloaded at: https://www.mdpi.com/article/10.3390/biology11101538/s1, Figure S1: Negative controls of nervous system immunostaining in embryo and larvae of *A. japonicus* by incubating with 1xPBS to verify the specificity of the primary antibody. DAPI was used for nuclear staining (blue). (A) blastula; (B) gastrula; (C) late-gastrula; (D) auricularia; (E) doliolaria; (F) pentactula; Table S1: Primer sequences used in the RACE and qRT-PCR amplifications.; Data S1: The identity percentage of the hits for the results of tBLASTn-based analysis.
