# Supplementary material for: Nervous System Development and Neuropeptides Characterization in Embryo and Larva: Insights from a Non-Chordate Deuterostome, the Sea Cucumber Apostichopus japonicus
Source: Biology (Basel). 2022 Oct 20;11(10):1538. doi: 10.3390/biology11101538 (PMC9598280; doi:10.3390/biology11101538)
Supplement: Supplementary file 1 [file biology-11-01538-s001.zip › Legends for supplementary materials.pdf]

Supplementary Table S1. Primer sequences used in the RACE and qRT-PCR amplifications.

Supplementary Figure S1. Negative controls of nervous system immunostaining in embryo and larvae of *A. japonicus* by incubating with 1xPBS to verify the specificity of the primary antibody. DAPI was used for nuclear staining (blue). (A) blastula; (B) gastrula; (C) late-gastrula; (D) auricularia; (E) doliolaria; (F) pentactula.

Supplementary Data S1. The identity percentage of the hits for the results of tBLASTn-based analysis.
