# Supplementary material for: Nervous System Development and Neuropeptides Characterization in Embryo and Larva: Insights from a Non-Chordate Deuterostome, the Sea Cucumber Apostichopus japonicus
Source: Biology (Basel). 2022 Oct 20;11(10):1538. doi: 10.3390/biology11101538 (PMC9598280; doi:10.3390/biology11101538)
Supplement: Supplementary file 1 [file biology-11-01538-s001.zip › Supplementary Table S1.pdf]

Supplementary Table S1. Primer sequences used in the RACE and qRT-PCR amplifications.

Gene name	Primer sequences (5' → 3')	Usage
AjPPLN2P	TTTGTCCTGGCGGCCTGTGTATTGA	RACE
AjTRHP	CGGGAGGAGACCTAGATGGCCTGGA	
AjMS21P	ACGAGAACCCTGTTGCTGTTTGTGTGCC	
AjCTP	CGAGCTGTGTCGATAACATTACGCATGG	
AjHolotocinP	TGGCTAGGCTTTCAGTGTGTGGAGT	
AjPPLN2P-F	AGAGACGCAGAGAAGAGATT	qRT-PCR
AjPPLN2P-R	TCTTGCCGCCTATCATACT	
AjTRHP-F	GGACAAGCAATGGATGACAA	
AjTRHP-R	ATCACCTCCTGGCAGTTG	
AjMS21P-F	ACCCTGTTGCTGTTTGTG	
AjMS21P-R	CATCATTCCTGGCTCTACC	
AjCTP-F	GTTTGGCGGTACCCATTAC	
AjCTP-R	GGCGTTCGGTCTGTTAATAG	
AjHolotocinP-F	ATGTGTTCTGTGCCGCTCTT	
AjHolotocinP-R	GCAATGGTCTCCTACGACTTCA	
AjKPP-F	CTGTCATTGCTCTGTGGAA	
AjKPP-R	GGTCATCTTCGTCTTGTTCT	
AjOXP1-F	CCTCCTCCTCCTCAACAT	
AjOXP1-R	GTGCTCGTCGTGATAGTC	
AjOXP2-F	TTGTTCCAAGATCCGTGATT	
AjOXP2-R	TTCCAGCGTATGTCCGAT	
AjGnRHP-F	AGGACGGACATCAACTCTG	
AjGnRHP-R	GGACTCTATCATCGAAGCAAT	
AjBAP-F	CGTCAAGCAGATTCAGCAA	
AjBAP-R	TGTTAGCGTTACCTTCACTT	
β-Actin-F	AAGGTTATGCTCTTCCTCACGCT	
β-Actin-R	GATGTCACGGACGATTTCACG	
β-Tubulin-F	GAAAGCCTTACGACGGAACA	
β-Tubulin-R	CACCACGTGGACTCAAAATG	
